# Potential new biomarkers for endometrial cancer

**DOI:** 10.1186/s12935-019-0731-3

**Published:** 2019-01-21

**Authors:** Michelle H. Townsend, Zac E. Ence, Abigail M. Felsted, Alyssa C. Parker, Stephen R. Piccolo, Richard A. Robison, Kim L. O’Neill

**Affiliations:** 1 Department of Microbiology and Molecular Biology, Brigham Young University, 3142 LSB, Provo, UT 84602 USA; 2 Department of Biology, Brigham Young University, Provo, UT USA; 3 Department of Biomedical Informatics, University of Utah, Salt Lake City, UT 84132 USA

**Keywords:** AURKA, Biomarkers, Endometrial cancer, HPRT1, JAG2, PGK1

## Abstract

**Background:**

Incidence of endometrial cancer are rising both in the United States and worldwide. As endometrial cancer becomes more prominent, the need to develop and characterize biomarkers for early stage diagnosis and the treatment of endometrial cancer has become an important priority. Several biomarkers currently used to diagnose endometrial cancer are directly related to obesity. Although epigenetic and mutational biomarkers have been identified and have resulted in treatment options for patients with specific aberrations, many tumors do not harbor those specific aberrations. A promising alternative is to determine biomarkers based on differential gene expression, which can be used to estimate prognosis.

**Methods:**

We evaluated 589 patients to determine differential expression between normal and malignant patient samples. We then supplemented these evaluations with immunohistochemistry staining of endometrial tumors and normal tissues. Additionally, we used the Library of Integrated Network-based Cellular Signatures to evaluate the effects of 1826 chemotherapy drugs on 26 cell lines to determine the effects of each drug on HPRT1 and AURKA expression.

**Results:**

Expression of HPRT1, Jag2, AURKA, and PGK1 were elevated when compared to normal samples, and HPRT1 and PGK1 showed a stepwise elevation in expression that was significantly related to cancer grade. To determine the prognostic potential of these genes, we evaluated patient outcome and found that levels of both HPRT1 and AURKA were significantly correlated with overall patient survival. When evaluating drugs that had the most significant effect on lowering the expression of HPRT1 and AURKA, we found that Topo I and MEK inhibitors were most effective at reducing HPRT1 expression. Meanwhile, drugs that were effective at reducing AURKA expression were more diverse (MEK, Topo I, MELK, HDAC, etc.). The effects of these drugs on the expression of HPRT1 and AURKA provides insight into their role within cellular maintenance.

**Conclusions:**

Collectively, these data show that JAG2, AURKA, PGK1, and HRPT1 have the potential to be used independently as diagnostic, prognostic, or treatment biomarkers in endometrial cancer. Expression levels of these genes may provide physicians with insight into tumor aggressiveness and chemotherapy drugs that are well suited to individual patients.

## Background

Endometrial cancer is the fourth most common cancer in women with 12,990 new diagnoses and 4120 deaths in 2016 in the United States [1]. Over 710,200 women are living with endometrial cancer in the United States, and approximately 2.8% of women will be diagnosed with the disease at some point during their lifetime. As the most significant risk factor for endometrial cancer is obesity, a majority of the biomarkers used to detect and monitor endometrial cancer development are related to metabolic and endocrine alterations [2]. Androgens, estrogens, prolactin, thyroid stimulating hormone, leptin, and adiponectin are a few of the biomarkers utilized to highlight risk of endometrial cancer development. While these biomarkers can be useful, they are oftentimes somewhat subjective as the levels of these hormones fluctuate naturally, are generally elevated with obesity, and are not necessarily unique to cancer development [2, 3]. In order to find new biomarkers that may act as diagnostic biomarkers for endometrial cancer, we evaluated Jagged2 (JAG2), Aurora Kinase A (AURKA), Phosphoglycerate Kinase 1 (PGK1), and Hypoxanthine Guanine Phosphoribosyltransferase 1 (HPRT1) for their role in cellular proliferation and cancer development. We evaluated these genes because of their upregulation and diagnostic potential in other cancer types [4–9].

JAG2 is a notch transmembrane ligand. Notch signaling is a conserved signaling pathway linked to the development of several cancers due to its role in cell fate, cellular proliferation regulation, and cell death [10]. This is exemplified by the fact that Notch signaling regulates stem cell proliferation and differentiation [11]. Within cancer, Notch signaling mediates hypoxia, invasion, and chemoresistance [12], and JAG2 expression in primary tumors has been correlated with vascular development and angiogenesis [13]. In addition, elevated levels of JAG2 result in significant chemoresistance, and when JAG2 is knocked down in mice, tumor cells become sensitive to chemotherapeutics (doxorubicin) [8]. Notch signaling has been identified as an important pathway for carcinogenesis of the endometrium [14]. Additionally, JAG2 has been shown to be a promising target in several cancer cell lines, as specific antibody–drug conjugates have resulted in tumor reduction [15].

AURKA is a cell-cycle regulated kinase that functions in spindle formation and chromosome segregation during the M phase of the cell cycle. AURKA has been shown to be a downstream target of MAPK1, which is a major force in cellular proliferation in several cancer cells [16]. The protein is also elevated in a variety of cancers and has a significant association with disease recurrence [6, 7]. Because AURKA is upregulated in cancers, efforts have been made to target the protein to aid in tumor reduction. Upon AURKA suppression, cancer cells become sensitive to chemotherapeutics and overall tumor growth is suppressed in a variety of cancer cells (docetaxel and taxane) [17, 18]. The role AURKA may play as a diagnostic biomarker in endometrial cancer has not been well studied, although it has shown promising results in other cancer types [6, 7, 19–21].

PGK1 is involved in the glycolysis pathway and functions by transferring a phosphate group from 1,3-bisphosphoglycerate to ADP to form ATP [22, 23]. As an enzyme involved in generating valuable energy for the cell, especially in hypoxic conditions, PGK1 has been correlated with cancer development and progression in a variety of tumor types [9, 24, 25]. Its role in promoting tumor proliferation is linked to PGK1’s ability to promote tumor angiogenesis [26, 27], DNA replication and repair [28, 29], and cancer metastasis [25, 30]. While the protein is elevated internally in several cancers, it is also actively secreted from tumor cells, where it cleaves plasminogen to create angiostatin [31]. PGK1 has been shown to be upregulated in several cancer types, but has not been evaluated for upregulation in endometrial cancer [25, 32].

HPRT1 is a nucleotide salvage enzyme involved in the cell cycle [33, 34]. This enzyme is a transferase responsible for producing guanine and inosine nucleotides by transferring a phosphoribose from PRPP to guanine and inosine bases, respectively, during cellular maintenance [35, 36]. As cells rapidly divide, the need for nucleotides increases, and subsequently HPRT1, has been shown to be elevated in several malignant settings [4, 37]. As the enzyme shows upregulation in malignant tissue while maintaining stable levels in normal tissue, it has the potential to be used as a biomarker for cancer development in several cancer types.

We decided to evaluate these enzymes in endometrial cancer because they have all shown promising diagnostic potential in other tissue types as biomarkers for disease development and progression but have not been evaluated in endometrial cancer. As malignant endometrial biomarkers are less established, we hope to identify additional markers for malignancy to aid in the early diagnosis and possible treatment of endometrial cancer.

## Materials and methods

### Chemicals/reagents

DIVA Decloaker 10x, Background Sniper, Mach 4 HRP polymer, DAB Peroxidase, Hematoxylin, Hydrophobic pen, and Universal Negative antibodies were all obtained from Biocare Medical, Concord, CA. Anti-JAG2 (LifeSpan Biosciences, Inc. Seattle, USA), Anti-AURKA (Sigma-Aldrich, St. Louis, USA), and anti-PGK1 (Abcam, Cambridge, UK) were stored at − 20 °C. Anti-HPRT monoclonal antibody (Abcam, Cambridge, UK) was aliquoted and stored at − 20 °C. GAPDH polyclonal antibody (Cell signaling) was aliquoted and stored at − 20 °C. Tween20 (Fisher Reagents, Waltham MA) was stored at room temperature. Hydrogen Peroxide at 30% (Fisher Reagents, Waltham MA) was stored at 4 °C.

### Tissue microarray samples

Tissue microarrays were obtained from Biomax and stained for GAPDH, HPRT, JAG2, AURKA, PGK1, and with an isotype control. Patients were all female and ranged in age from 21 to 63. Normal (n = 9), cancer adjacent (n = 9), and malignant tissue (n = 54) (grade 1–3) were included in the analysis (Table 1).

**Table 1:** Protein expression within patient tissue

Protein	n	General function	Average gray value malignant	Average gray value CAT	Average gray value normal
HPRT	68	Nucleotide salvage	157.206	186.176	223.207
PGK1	71	Glycolytic enzyme	107.273	154.437	171.748
AURKA	72	Cycle-regulated kinase	209.994	236.147	244.352
Jag2	72	Protein coding	143.635	194.297	186.269

*CAT* cancer adjacent tissue

### Immunohistochemistry

Protein levels were assessed using protocols described by Townsend et al. with slight modifications [4]. Briefly, tissues were rehydrated, washed, and treated with DIVA Decloaker. Following a hydrogen peroxide wash, tissues were treated with a Background Sniper followed by a primary antibody (1:100 dilution). After a series of washes, the tissues were treated with DAB Peroxidase and hematoxylin and imaged using a standard light microscope.

### Tissue quantification

ImageJ software was utilized to quantify staining intensity [38]. An IHC toolbox plugin was selected with the “DAB (more brown)” option to remove staining that did not result from DAB. After this modification, the images were converted to a grayscale and a threshold was applied to eliminate areas of negative space that could potentially bias the results. Once a universal threshold was applied, the average gray intensity of the tissue was collected.

### Tumor gene-expression analysis

We obtained RNA-sequencing and clinical outcomes data for Uterine Corpus Endometrial Carcinoma (UCEC) samples from The Cancer Genome Atlas (TCGA) [39]. We used transcripts-per-million values, summarized at the gene level. These data were derived from tumor and normal samples.

Survival was calculated using a Cox proportional hazard model. In addition to gene expression (primary variable), covariates included gene expression and clinical factors such as age, race, and tumor purity. Kaplan–Meier curves were generated to compare survival of patients with the highest 20% of target gene expression against those with the lowest 20% of target gene expression. The statistical analyses and curve generations were calculated utilizing the TIMER program developed by Li et al. [40].

### Drug response analyses

We evaluated the effects of chemotherapy treatments on cell lines using two publicly available databases. First, we examined data from the Cancer Cell Line Encyclopedia (CCLE) [41]. We obtained treatment-response data for 24 drugs that were available from the CCLE portal and used the area above the fitted dose–response curve (ActArea) as a metric of treatment response [42]. We obtained transcript-level expression levels for CCLE [43] and summed protein-coding transcript values to gene-level values using a custom Python script (https://python.org). For each of four genes (HPRT1, AURKA, JAG2, and PGK1), we identified cell lines for which drug-response and gene-expression data were available and then ranked the cell lines according to expression of the respective genes. Next, we selected the lowest- and highest-expressing cell lines for each gene and used a Mann–Whitney U test to evaluate differences in ActArea values between these cell-line groups. To perform these calculations, we used the R statistical software (version 3.4.3) [44].

Second, we evaluated data from the Library of Integrated Network-based Cellular Signatures, which contains gene-expression profiles for cell lines after drug perturbations. We wrote a Python (version 3.6.5) script to extract HPRT1 and AURKA expression values from the LINCS database for samples from 26 cell lines for which data were available. We used the Level 5 data, which were generated using the L1000 platform [45], normalized using a z-score methodology within each plate, and averaged across replicates. Using the R (version 3.4.4) [44] statistical software and the readr package (version 1.1.1) [46], we parsed the metadata file to identify experiments where the cell lines had been treated with chemotherapeutic compounds (pert_type = “trt_cp”). The summarized data values indicate relative gene-expression levels for cells treated with a given compound relative to control-treated cells. To perform this filtering and data transformation, we used the dplyr (version 0.7.4) [47] and reshape2 (version 1.4.3) packages [48]. Before plotting the data, we grouped the values for each cell line by compound name. We identified the median value for each group and sorted the values from lowest to highest. Then we used the superheat package (version 1.0.0) to create heatmaps with data from the 7 cell lines with the most treatment data [49]. The code and data we used for this analysis can be found at https://bitbucket.org/alyssaparker99/lincs-heatmaps.

### Statistical analysis

Staining intensities between tissue samples were analyzed using an ANOVA test with the multiple comparison method. Additionally, unpaired *t* tests were utilized in conjunction to confirm statistical significance. These statistical tests were performed in GraphPad Prism 7 software. Differences were considered significant when the p value was < 0.05. Asterisks were used in figures to indicate levels of significance with ns = p > 0.5, *p ≤ 0.05, **p ≤ 0.01, ***p ≤ 0.001, and ****p ≤ 0.0001.

## Results

### JAG2, AURKA, PGK1, and HPRT1 had significant upregulation in malignant samples when compared to normal

**Fig. 1:**
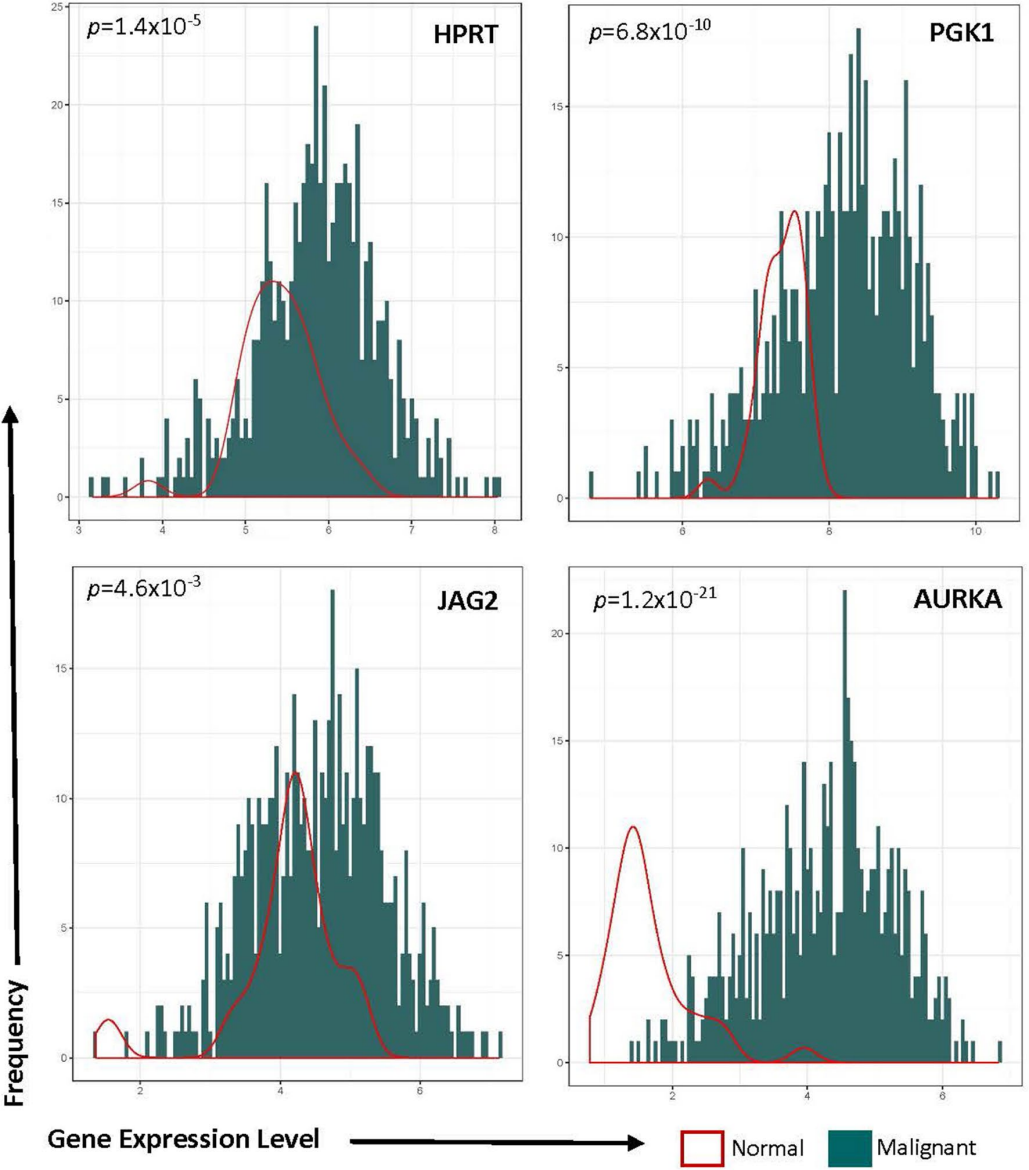
Gene expression in patient samples. HPRT, PGK1, JAG2, and AURKA were analyzed for gene expression in both normal (red line) and malignant (blue histogram) samples. Relative protein expression is quantified on the X-axis (represented as transcripts per million), while the frequency of the expression is plotted on the Y-axis

We evaluated gene-expression levels for AURKA, JAG2, HPRT1, and PGK1 in tumors and normal tissues from TCGA. Upon comparing malignant and normal samples, we observed a consistent elevation of each of the genes in malignant tissues (Fig. 1). JAG2 had the smallest elevation overall (*p*-value = 4.6 × 10^−3^), while AURKA showed the largest increase (p-value = 1.2 × 10^−21^). This upregulation indicates that these genes may be useful as diagnostic markers of endometrial cancer, as they have differential expression between normal and malignant samples.

**Fig. 2:**
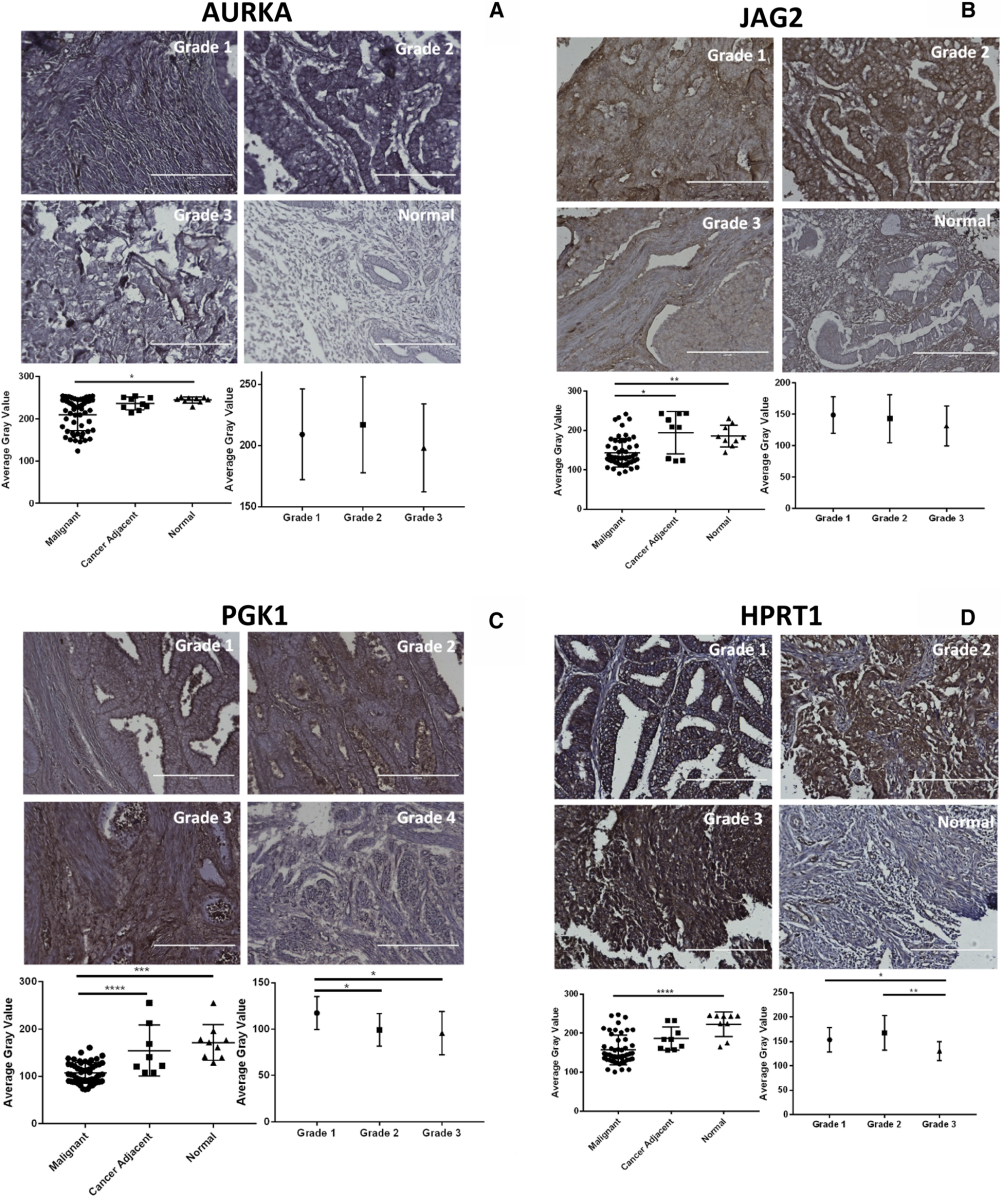
Tissue evaluation of AURKA, JAG2, PGK1, and HPRT. Tissues were quantified on a gray scale with lower values indicating darker staining intensity. **a** AURKA expression and **b** JAG2 expression was significant between malignant and normal samples, but showed no significance between cancer grade. **c** PGK1 expression and **d** HPRT expression showed significance both between normal and malignant samples in addition to between cancer grade

**Fig. 3:**
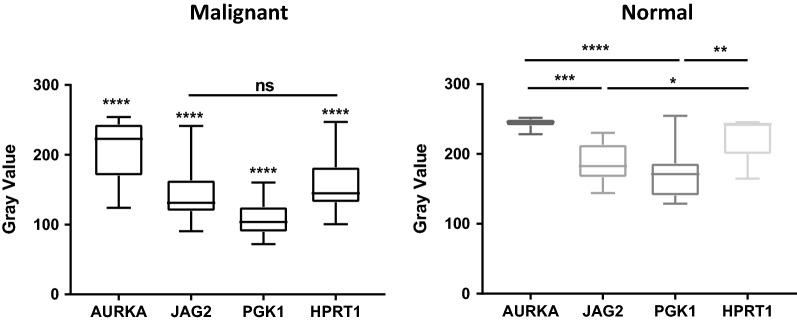
Gene expression between normal and malignant patient samples. Tissues were quantified on a gray scale with lower values indicating darker staining intensity. Across malignant samples, patients exhibited a variety of expression of each of the genes evaluated that were all significant from each other with the exception of JAG2 and HPRT expression. In addition, normal samples also showed a variety of expression of the genes, with PGK1 showing the highest expression and AURKA showing the lowest expression

When analyzing protein levels in tissue microarrays from a separate cohort, we again found that all four genes were significantly elevated within malignant samples (Fig. 2). This confirmed the initial analysis with gene expression data. In addition, we found that PGK1 and HPRT1 both showed significant differences between grades as there was a stepwise elevation of protein expression corresponding to grade. This indicates that HPRT1 and PGK1 may have a grade dependency, and could serve as biomarkers for tumor aggressiveness. All four genes showed a range of protein expression in both malignant and normal samples (Fig. 3).

**Fig. 4:**
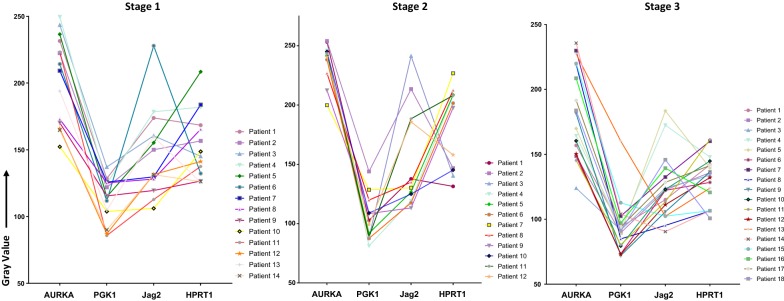
Individual patient expression of biomarkers. Each biomarker and their relative expression is plotted according to the patient. Relative expression is represented on the Y-axis, while the protein evaluated is represented on the X-axis. Individual patients did not show consistent biomarker elevation in any of the stages evaluated

To determine whether elevated expression of these genes occurred in the same patients, we plotted expression values for each patient jointly for all four genes. There was no pattern of concordant elevation across PGK1, AURKA, JAG2, and HPRT1. For example, patients with elevated levels of AURKA did not share the same high levels of HPRT1 or of any of the other genes (Fig. 4). This was observed in all cancer stages. For example, there were cases where the patient with the lowest expression of AURKA (patient 7 in Stage 2) also had the highest expression of HPRT1. This indicates that these biomarkers may be useful in identifying different patients and that each biomarker may be independently used to benefit further characterization of individual patient cancer types.

### AURKA and HPRT1 elevation have a significant impact on patient survival

**Fig. 5:**
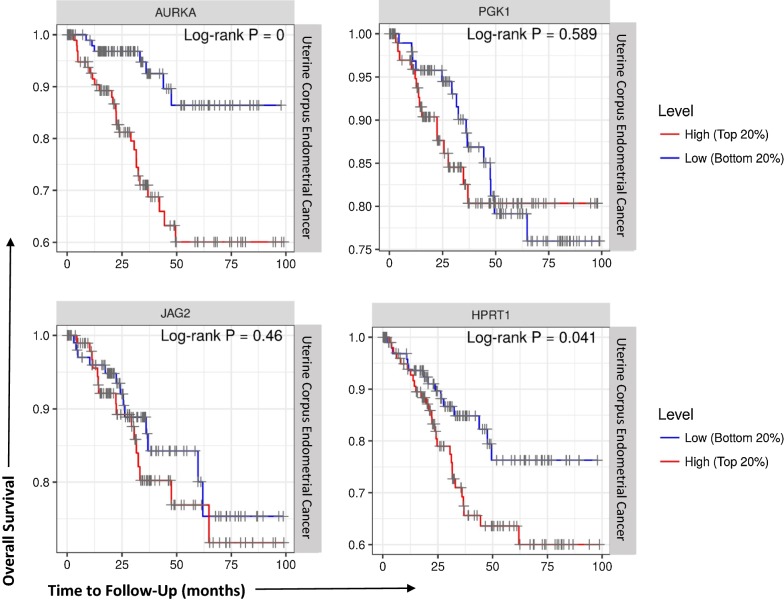
Survival of patients with elevated levels of JAG2, AURKA, PGK1, and HPRT1. We plotted the survival of patients with the highest 20% expression of each respective biomarker (red line) and compared them to the patients with the lowest 20% expression (blue line) over the course of 100 months. We found no statistically significant difference in survival with regards to high and low expression of PGK1 or JAG2, but found significant decreases in survival in patients with an elevation of AURKA (*p*-value < 0.0001) and HPRT1 (*p*-value = 0.041)

We evaluated overall patient survival in patients with the highest 20% of biomarker expression and patients with the lowest 20% biomarker expression to determine whether the elevation of these genes had any impact on survival. Both PGK1 (*p*-value = 0.589) and JAG2 (*p*-value = 0.46) showed insignificant differences between survival over the course of 100 months between high and low expressors. While there may have been elevation of these genes within cancer, they did not seem to contribute to survival outcomes. Interestingly, both AURKA and HPRT1 showed significant differences between survival in high vs low expressing patients. Following 100 months, patients with the highest 20% of AURKA expression showed significant (*p*-value < 0.0001) decreases in survival and AURKA elevation correlated with lower survival rates (Fig. 5). This same pattern was also observed for patients with elevated HPRT1 expression, as patients with the highest 20% HPRT1 expression had significantly (*p*-value = 0.041) decreased survival compared to their lower expressing counterparts. This shows that both AURKA and HPRT1 may have significance beyond diagnostic; they also may be useful, as prognostic biomarkers for uterine corpus endometrial cancer.

### Drug treatments of cell lines with high and low target gene expression

**Fig. 6:**
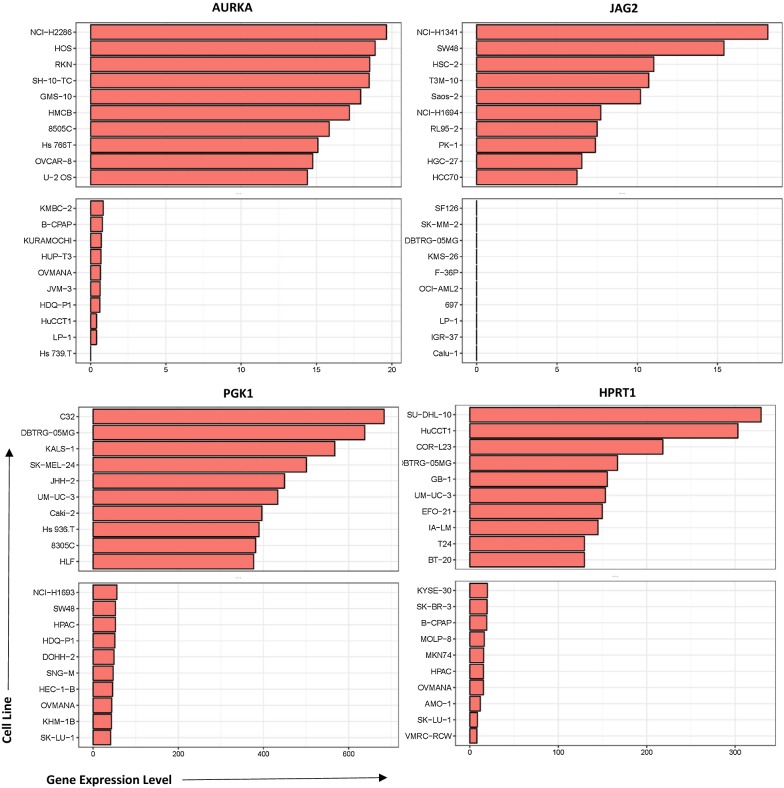
Cell lines ranked by their relative expression of JAG2, AURKA, PGK1, and HPRT1. Cell lines were ranked according to their gene expression level (transcripts per million) and the 10 highest expressing and 10 lowest expressing cell lines are shown

**Fig. 7:**
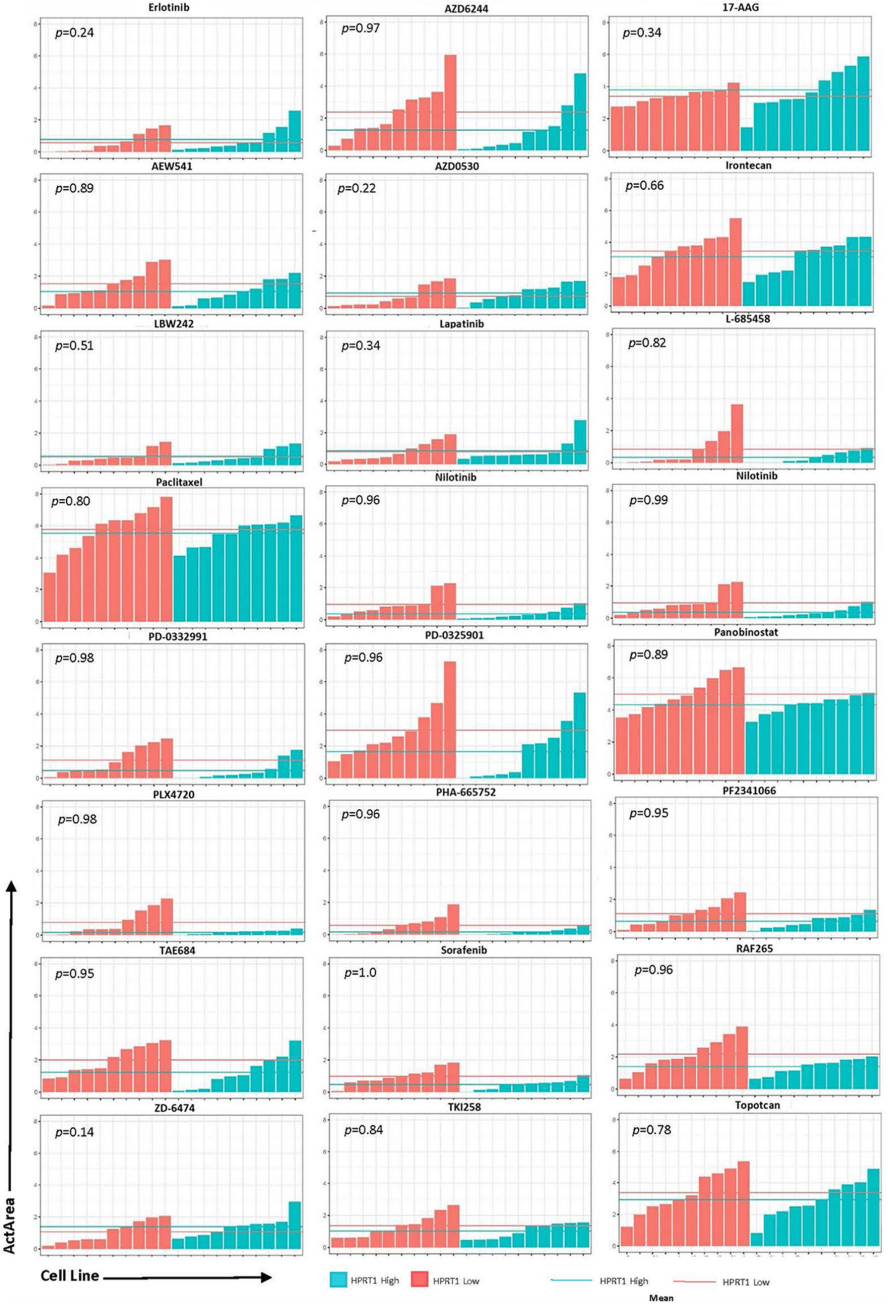
Drug responses of cell lines with elevated JAG2. The 20 cell lines with the highest and lowest expression for each target gene from the previous analysis in Fig. 6 (X-axis) were evaluated via their Activity Area (ActArea) in response to drug treatments. Drug responses are represented by individual graphs with the mean ActArea plotted on the Y-axis. Drugs with a high ActArea indicate sensitivity, while drugs with a low ActArea indicate resistance. The mean ActArea is represented by a line within the figure to indicate the average increase or reduction between the high expressing and low expressing cell lines

**Fig. 8:**
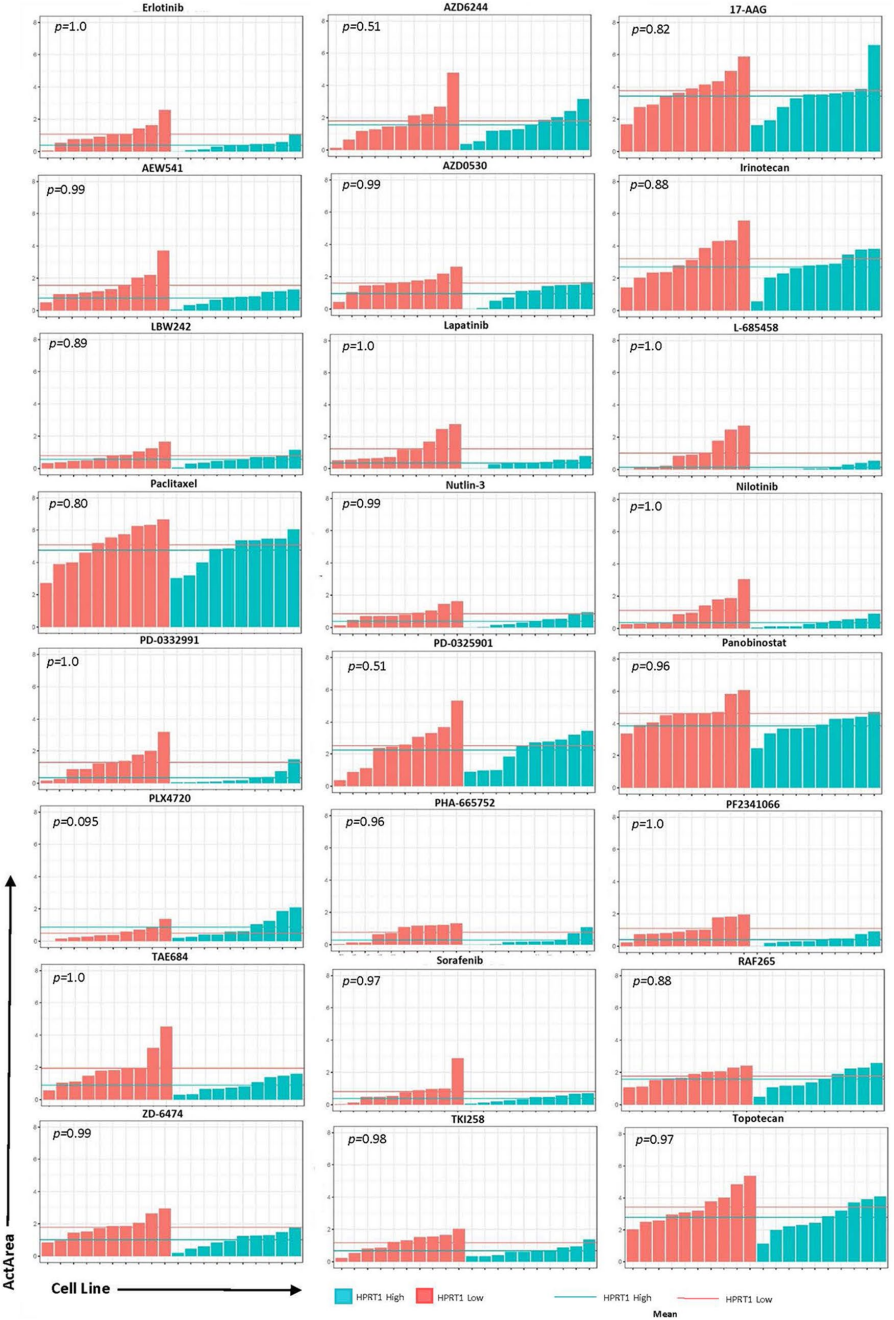
Drug responses of cell lines with elevated PGK1. The 20 cell lines from the previous analysis in Fig. 6 were evaluated via their ActArea in response to drug treatments. The mean ActArea is represented by a line within the figure to indicate the average increase or reduction between the high expressing and low expressing cell lines

**Fig. 9:**
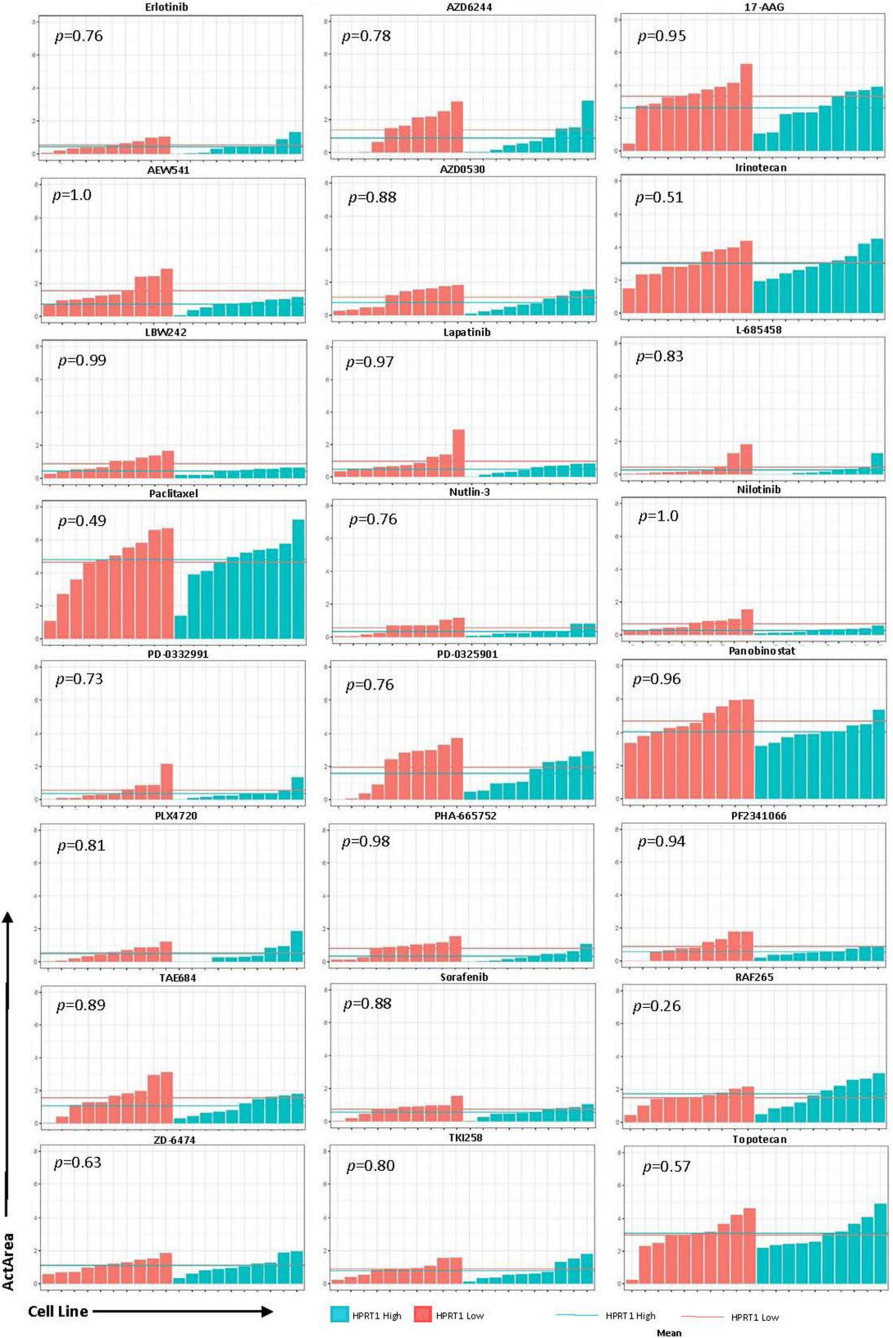
Drug responses of cell lines with elevated HPRT1. The 20 cell lines from the previous analysis in Fig. 6 were evaluated via their ActArea in response to drug treatments. The mean ActArea is represented by a line within the figure to indicate the average increase or reduction between the high expressing and low expressing cell lines

**Fig. 10:**
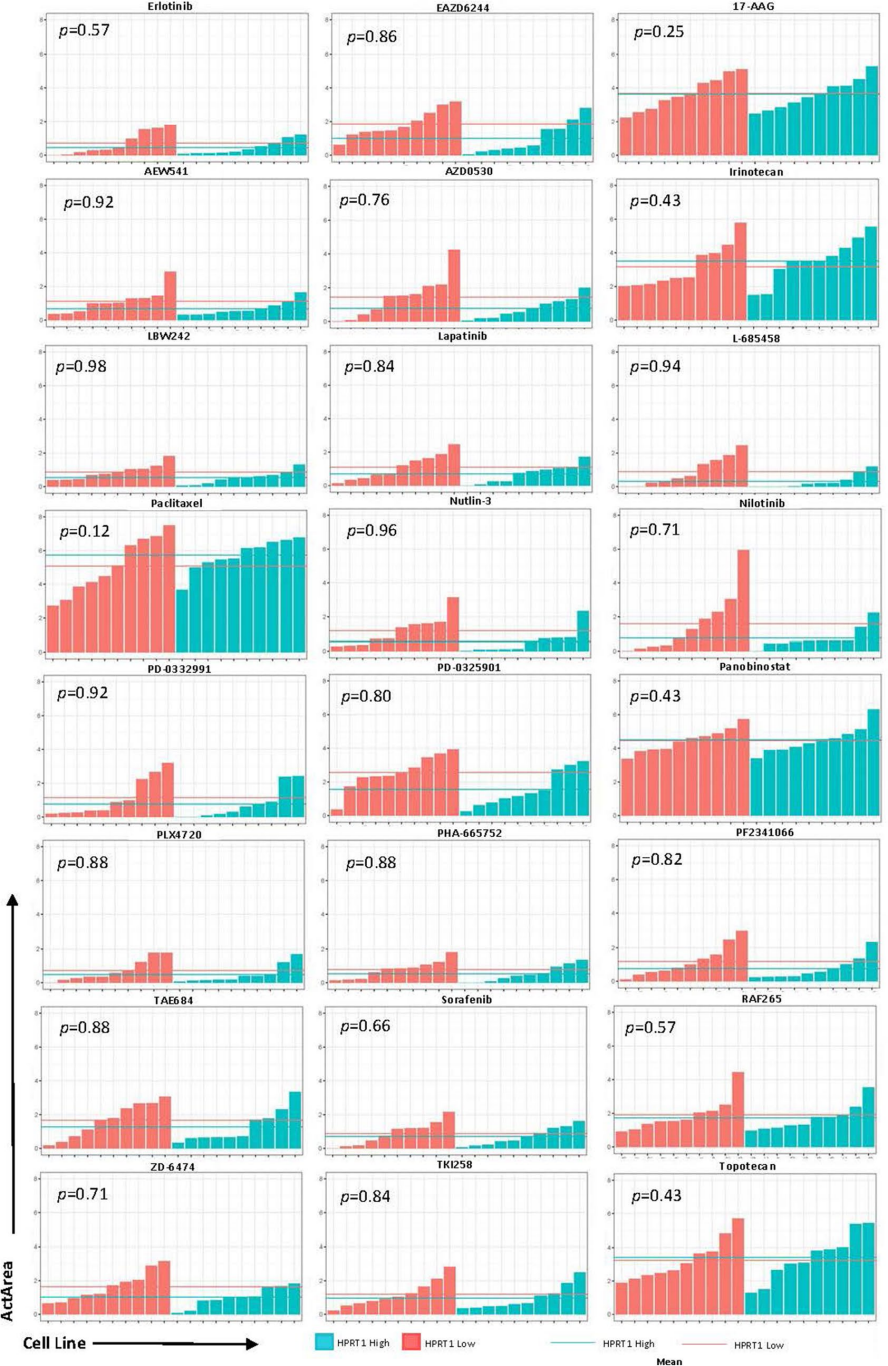
Drug responses of cell lines with elevated AURKA. The 20 cell lines from the previous analysis in Fig. 6 were evaluated via their ActArea in response to drug treatments. The mean ActArea is represented by a line within the figure to indicate the average increase or reduction between the high expressing and low expressing cell lines

To determine whether these genes could be utilized as biomarkers for physicians when deciding treatment options, we analyzed the effects of 24 drugs on cell lines with relatively high and low expression of AURKA, JAG2, PGK1, and HPRT1. Cell lines were ranked according to their expression of each gene and highest and lowest expressing cell lines were chosen for analysis (Fig. 6). Although there was no significance observed, there were some responses that appeared to have a larger impact than others. Drugs with the largest differences were PD-0325901 (MEK inhibitor), TAE684 (ALK inhibitor), AEW541 (IGF-1R inhibitor), and Nilotinib (tyrosine kinase inhibitor) in JAG2, PGK1, HPRT1, and AURKA, respectively. Several of the drug responses were negligible as the mean ActArea was almost identical in a majority of the responses between the high and low expression cell lines (Figs. 7, 8, 9, 10).

### Drugs with the largest impact on AURKA and HPRT1 expression

**Fig. 11:**
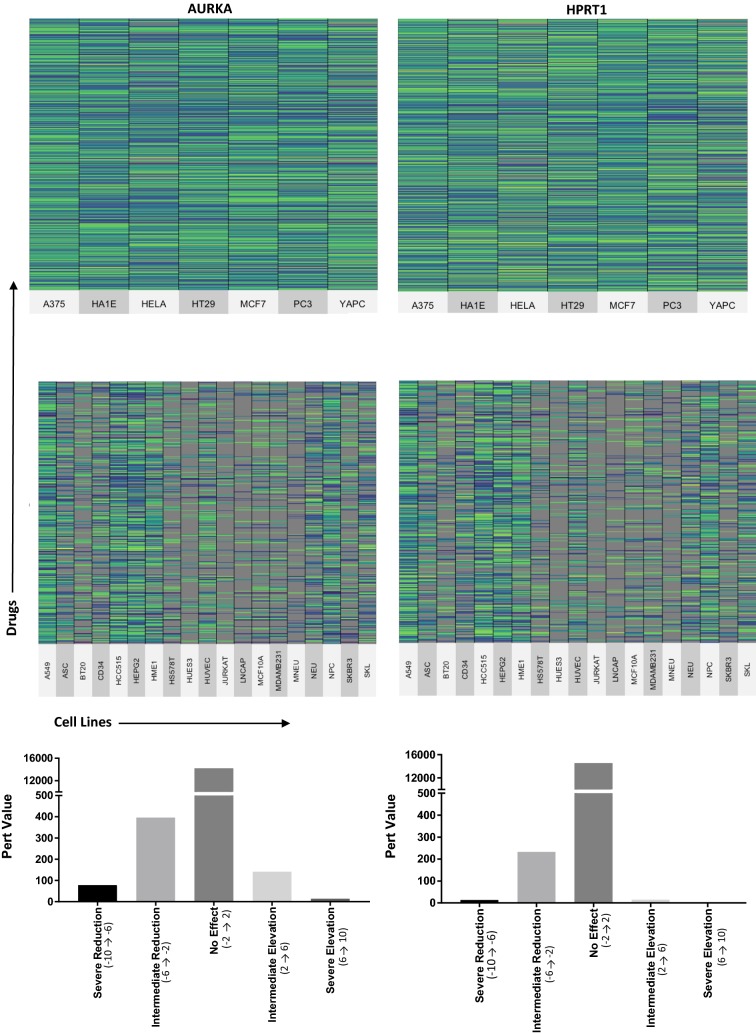
Drugs that lower the expression of JAG2, HPRT1, AURKA, and PGK1. Cell lines (x-axis) were evaluated for their expression of AURKA and HPRT1 pre and post treatment with drugs (y-axis). The relative changes in protein expression are indicated by the heat map legend and show the variety of responses to various drugs. The events and their effects on target gene expression are indicated by the bar graphs

As HPRT1 and AURKA elevation showed prognostic significance (Fig. 5), we analyzed data from the LINCS, a publicly available resource that contains gene-expression response signatures for 1826 chemotherapy drugs and 26 cell lines. We searched for drug treatments that caused significant declines in HPRT1 and/or AURKA expression. These responses varied widely across drug treatments and cell lines with some drugs increasing the expression of the genes and, others decreasing expression. The vast majority of drug treatments had no impact on HPRT1 or AURKA expression. We focused on seven cell lines for which data were most available (Fig. 11). For both genes, over 12,000 drug-cell line interactions resulted in no effect. When evaluating AURKA expression, 78 interactions resulted in a severe reduction, 396 resulted in an intermediate reduction, while 14 resulted in a severe elevation and 141 resulted in an intermediate elevation of the gene. When evaluating HPRT1 expression, 13 interactions resulted in a severe reduction, 233 resulted in an intermediate reduction, while 15 resulted in an intermediate elevation of the gene (Table 2). This indicates that AURKA may be a better prognostic biomarker than HPRT1 as there is a larger number of events where the protein was significantly decreased upon treatment.

**Table 2:** Impact of drug treatment on AURKA and HPRT expression

	Description	PTEZ score range	# samples
AURKA	Severe reduction	− 10 → − 6	78
	Intermediate reduction	− 6 → − 2	396
	No effect	− 2 → 2	14,174
	Intermediate elevation	2 → 6	141
	Severe elevation	6 → 10	14
HPRT1	Severe reduction	− 10 → − 6	13
	Intermediate reduction	− 6 → − 2	233
	No effect	− 2 → 2	14,553
	Intermediate elevation	2 → 6	15
	Severe elevation	6 → 10	0

*PTEZ* post-treatment expression z-score

The 10 drugs that produced the largest reduction in AURKA expression were Ro-4987655, Genz-644282, OTS-167, Vorinostat, Pralatrexate, Epirubicin, Ro-4987655, Pralatrexate, JNJ-26481585, and R-547. Each of these drugs has a different mechanism of action but most were involved in DNA synthesis and regulation. Of note, when analyzing the drugs that resulted in an increase in AURKA expression, we found that 9 of 10 drugs were directly involved in inhibiting microtubule function or inhibited PLK. This was consistent throughout our analysis and indicates AURKA may be connected in a regulatory fashion to these cellular mechanisms (Table 3).

**Table 3:** Effective drugs for the reduction of AURKA

Cell line	Drug	Inhibition target	Target symbol	PTEZ score
Drugs with significant reduction in AURKA expression post treatment				
A375	Ro-4987655	Mitogen-activated protein kinase	MEK	− 10
A375	Genz-644282	Topoisomerase I	Topo I	− 10
HUES3	OTS-167	Maternal embryonic leucine-zipper kinase	MELK	− 10
HUES3	Vorinostat	Histone deacetylase	HDAC	− 10
A375	Pralatrexate	DNA synthesis	–	− 9.838
MCF7	Epirubicin	Topoisomerase II	Topo II	− 9.471
HT29	Ro-4987655	Mitogen-activated protein kinase	MEK	− 9.284
MCF7	Pralatrexate	Metabolic	–	− 9.259
A375	JNJ-26481585	Histone deacetylase	HDAC	− 9.206
HT29	R-547	Cyclin dependent kinase	CDK	− 8.938
Drugs with an increase in AURKA expression post treatment				
PC3	BIIB-021	Heat shock protein 90	HSP90	6.298
HT29	NMS-1286937	Polo-like kinase 1	PLK	6.407
HELA	NMS-1286937	Polo-like kinase 1	PLK	6.426
HT29	Docetaxel	Microtubule function	–	6.458
HT29	Epothilone-b	Microtubule function	–	6.518
HT29	Indibulin	Microtubule function	–	6.552
HELA	Dolastatin-10	Microtubule function	–	6.666
HELA	Volasertib	Polo-like kinase 1	PLK	6.732
HT29	Epothilone-b	Microtubule function	–	6.898
HELA	Combretastatin-A-4	Microtubule function	–	7.007

*PTEZ* post-treatment expression z-score

Drugs that resulted in the highest reduction in HPRT1 expression were AS-703026, OTS-167, BGT-226, genz-644282, AS-703026, SN-38, SN-38, TAK-733, paclitaxel, and KX2-391. Of these, six were of either Topoisomerase I (Topo I) or MEK. This may indicate a relationship between HPRT1 regulation and regulation of Topo I or the MEK pathway (Table 4).

**Table 4:** Effective drugs for the reduction of HPRT1

Cell line	Drug	Inhibition target	Target symbol	PTEZ score
Drugs with significant reduction in HPRT1 expression post treatment				
HT29	AS-703026	Mitogen-activated protein kinase	MEK	− 9.822
HUES3	OTS-167	Maternal embryonic leucine-zipper kinase	MELK	− 9.707
Jurkat	BGT-226	Phosphoinositide 3-kinase	P13K	− 8.533
MCF7	genz-644282	Topoisomerase I	Topo I	− 7.601
A375	AS-703026	Mitogen-activated protein kinase	MEK	− 7.119
MCF7	SN-38	Topoisomerase I	Topo I	− 6.904
A375	SN-38	Topoisomerase I	Topo I	− 6.702
HT29	TAK-733	Mitogen-activated protein kinase	MEK	− 6.594
MCF7	Paclitaxel	Microtubule function	–	− 6.537
MCF7	KX2-391	Sarcome	Src	− 6.366
Drugs with an increase in HPRT1 expression post treatment				
MNEU	Dinaciclib	Cyclin dependent kinase	CDK	2.362
NPC	SB-939	Histone deacetylase	HDAC	2.39
MNEU	Mitoxantrone	Topoisomerase II	Topo II	2.412
NEU	Ischemin	P53 transcription	–	2.454
ASC	Mitoxantrone	Inflammation	–	2.551
NEU	NVP-BGJ398	Fibroblast growth factor receptor	FGFR	2.668
Jurkat	Tanespimycin	Heat shock protein	HSP	2.72
SKL	Dinaciclib	Cyclin dependent kinase	CDK	3.137
ASC	Dinaciclib	Cyclin dependent kinase	CDK	3.195
Jurkat	Dinaciclib	Cyclin dependent kinase	CDK	5.414

*PTEZ* post-treatment expression z-score

## Discussion

We have determined that there is a significant elevation of JAG2, HPRT1, AURKA, and PGK1 expression in endometrial cancer. With elevated expression upon malignancy, these genes can be utilized as a companion diagnostic tool to both identify and characterize endometrial cancer. As cancer specific biomarkers, these genes may serve as useful markers when analyzing endometrial cancer development within patient tissue. Additionally, HPRT and PGK1 show additional promise as possible biomarkers for cancer grade as the levels of the proteins elevated in a stepwise manner with higher cancer grade. These biomarkers have already shown utility in other cancer types [4–6, 8, 9, 16] and we have shown that their use may also extend to endometrial cancer.

While there are several epigenetic biomarkers for endometrial cancer (p52, KRAS, VEGF. PTEN, etc.) [50, 51], there remains a need to find suitable protein biomarkers for not only endometrial diagnosis, but also as possible targets for future therapies. Future directions to this work include evaluating a larger cohort of patients to determine whether the expression of these biomarkers could have clinical application. Especially in the case of both HPRT1 and AURKA, it may be beneficial to develop therapies to reduce their expression, thereby determining whether these genes play a critical role in cancer survival and proliferation as they show significant impact on overall patient survival.

In addition, the conserved pathways that HPRT1 and AURKA have in terms of drugs that inhibit or induce their expression, may indicate a regulatory relationship between the inhibited pathway and the proteins that have not yet been identified. The merit of this hypothesis is demonstrated as AURKA has a reciprocal regulation with PLK1 in mitotic entry and within spindle assembly [52]. This corresponds to the data we have observed as the drugs with the largest impact on AURKA elevation with the highest consistency are inhibitors of PLK1 and microtubule formation. Yet, the consistent relationship between drugs that inhibit HPRT1 expression are both inhibitors of Topo I and the MEK pathway. There has not been any investigation into the relationship between HPRT1 and these proteins/pathways and our initial data show that a possible link exists. With this in mind, this potential relationship merits further examination and could potentially elucidate novel gene interactions specific to cancer.

## Conclusions

We have determined genes with differential gene expression within endometrial cancer that also have a significant impact on overall patient survival. These biomarkers could be developed into a companion diagnostic tool in the identification and classification of endometrial cancer. In addition, they may aid in drug determination as certain drugs have a better response rate in patients with elevated levels of both AURKA and HPRT1.
